# Quantitative Comparison of the Hydration Capacity of Surface-Bound Dextran and Polyethylene Glycol

**DOI:** 10.1021/acs.langmuir.4c01582

**Published:** 2024-06-26

**Authors:** Chiara Perrino, Seunghwan Lee, Nicholas D. Spencer

**Affiliations:** † Laboratory for Surface Science and Technology, Department of Materials, Vladimir-Prelog-Weg 5, ETH Zurich, CH-8093 Zurich, Switzerland; ‡ Institute of Functional Surfaces, School of Mechanical Engineering, University of Leeds, LS2 9JT Leeds, U.K.

## Abstract

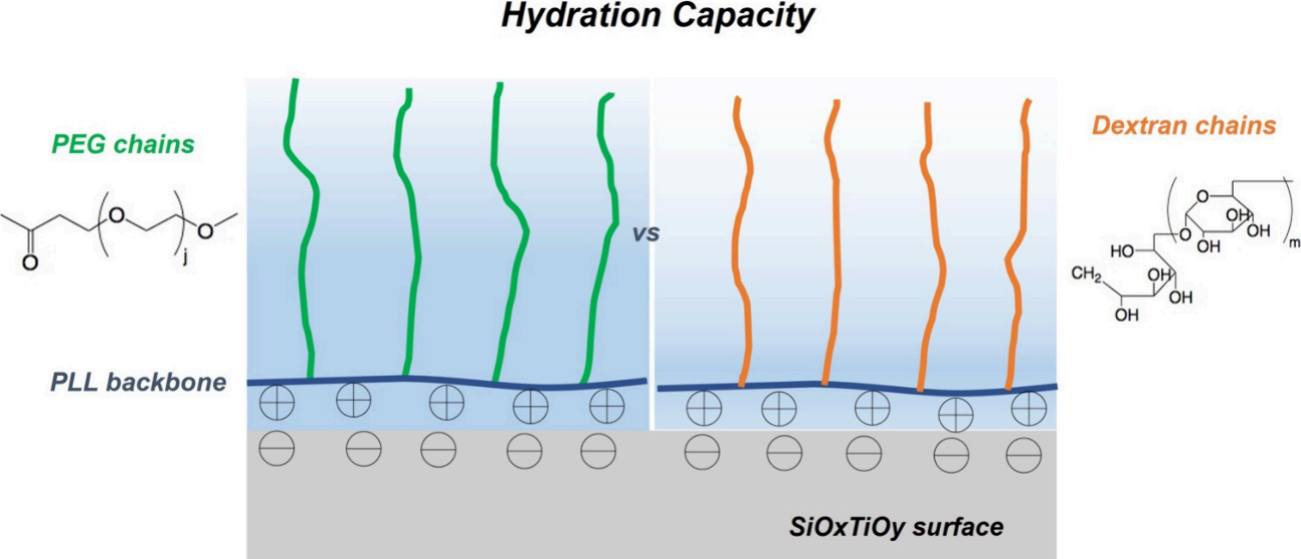

We have quantified and compared the hydration capacity (i.e., capability to incorporate water molecules) of the two surface-bound hydrophilic polymer chains, dextran (dex) and poly(ethylene glycol) (PEG), in the form of poly(l-lysine)-*graft*-dextran (PLL-*g*-dex) and poly(l-lysine)-*graft*-poly(ethylene glycol) (PLL-*g*-PEG), respectively. The copolymers were attached to a negatively charged silica–titania surface through the electrostatic interaction between the PLL backbone and the surface in neutral aqueous media. While the molecular weights of PLL and PEG were fixed, that of dex and the grafting density of PEG or dex on the PLL were varied. The hydration capacity of the polymer chains was quantified through the combined experimental approach of optical waveguide lightmode spectroscopy (OWLS) and quartz crystal microbalance with dissipation monitoring (QCM-D) to yield a value for areal solvation (Ψ), i.e., mass of associated solvent molecules within the polymer chains per unit substrate area. For the two series of copolymers with comparable stretched chain lengths of hydrophilic polymers, namely, PLL(20)-*g*-PEG(5) and PLL(20)-*g*-dex(10), the Ψ values gradually increased as the initial grafting density on the PLL backbone increased or as *g* decreased. However, the rate of increase in Ψ was higher for PEG than dextran chains, which was attributed to higher stiffness of the dextran chains. More importantly, the number of water molecules per hydrophilic group was clearly higher for PEG chains. Given that the −CH_2_CH_2_O– units that make up the PEG chains form a cage-like structure with 2–3 water molecules, these “strongly bound” water molecules can account for the slightly more favorable behavior of PEG compared to dextran in both aqueous lubrication and antifouling behavior of the copolymers.

## Introduction

**Figure 1:**
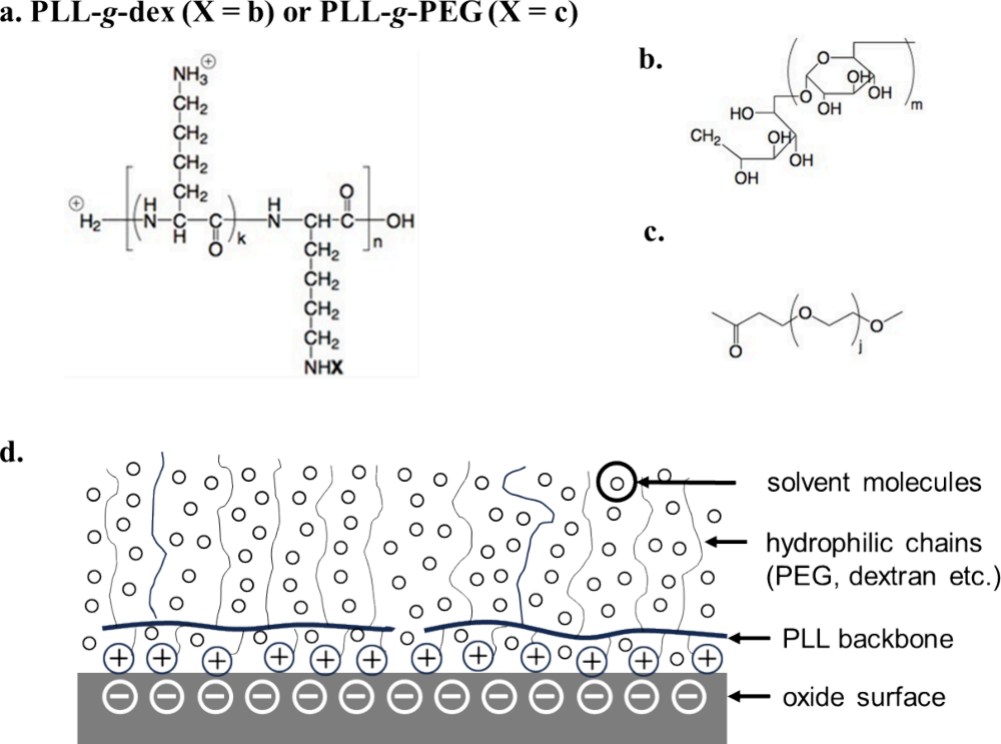
(a–c) The chemical structures of PLL-*g*-dex and PLL-*g*-PEG, dex, and PEG, respectively. (*k* should be taken as an average value. *k* + 1 represents the grafting ratio of the polymer.) (d) Schematic illustrating the adsorption of PLL-*g*-dex or PLL-*g*-PEG on oxide surfaces in aqueous media.

Surface-grafted, hydrophilic, brush-like polymers,^1−3^ including polyethylene glycol (PEG),^4−6^ polyelectrolytes,^7−9^ and glycans,^10−15^ represent examples of highly successful biomimicry of biological interfaces by polymeric coatings. They also provide a means for exploring various surface and interfacial modifications of the underlying substrates to promote properties such as antifouling and aqueous lubrication. Among many approaches to grafting hydrophilic polymer chains, copolymerization with primary amine units along the polycationic poly-l-lysine (PLL), to yield PLL-*g*-X copolymers (X = PEG,^16−19^ dextran,^20−24^ or poly(2-methyl-2-oxazoline),^25,26^ for example) (Figure 1), is unique.

This approach can provide stable anchoring onto the surface via multiple binding sites and already enables a degree of control of the grafting density of hydrophilic polymer chains at the polymer-synthesis step. In addition, the structural similarities of PLL-*g*-X copolymers allow for a comparison between different hydrophilic chains as to their structural and functional characteristics. PLL-*g*-dex is particularly noteworthy, as dextran (dex) is a naturally occurring glycan, and thus, the adsorbed copolymer resembles macromolecules of relevance to biointerfacial properties. Past studies on PLL-*g*-dex, which included variation of surface grafting density,^20−24^ often in comparison with PLL-*g*-PEG,^20,21^ revealed that dex chains display a highly stretched “brush-like” conformation as the distance between neighboring chains on the surface decreases but show slightly inferior performance in both antifouling^20^ and aqueous lubrication^21^ properties compared to their PEG-based counterparts. Discussion of the possible reasons for the differences between the surface-tethered polymer chains composed of dextran and PEG has been largely speculative. Factors suggested have included the higher flexibility of PEG chains compared to dextran’s bulkier sugar units,^27^ the chemical structure of dextran, which contains both hydrogen-bond acceptors and donors,^28^ the different conformation of the chains,^29,30^ and the different degree of hydration,^20,21^ but experimental investigation of these alternatives has been extremely rare to date.

In the present work, we focus on the quantitative measurement of the hydration capacity of PLL*-g*-dex and PLL*-g*-PEG copolymers, which might play an important role in determining their performance as lubricant additives and protein-resistant coatings. The amount of solvent adsorbed within PLL*-g*-PEG copolymers, and brush-like copolymers in general, has in fact been shown to be an important parameter affecting both their lubricating^16,31,32^ and protein-resistance behavior.^33,34^

PEG has been shown to form a unique structure with water; the water-binding properties of aqueous PEG solutions have been investigated experimentally by many experimental techniques, such as light scattering, neutron scattering, nuclear magnetic resonance (NMR), infrared spectroscopy, calorimetry, and molecular dynamics simulations as well.^35−41^ According to the most widely accepted models, hydrated PEG chains preferentially maintain a trans–gauche–trans (tgt) conformation, adopting a “cage-like” helical structure, such that the hydrophobic ethylene units (−CH_2_CH_2_−) are shielded from contacting water molecules, while the ether oxygen atoms undergo hydrogen bonding to the water molecules, with 2–3 water molecules binding per ethylene oxide unit.^36,37,42^

The solution properties of dextran and its interactions with water have also been investigated by many experimental techniques, such as light scattering, sedimentation velocity, viscosity measurements, and ultrasonic velocity measurements.^43−47^ Gekko et al.^45^ determined, for instance, the amount of bound water for dextran fractions with molecular weights below 50 kDa by means of sound-velocity measurements; the bound water expressed as molecules/OH group showed a decreasing trend (from 0.9 to ca. 0.5) upon increasing dextran molecular weight from 0.4 to 2 kDa and then stayed constant at 0.5 for higher molecular weights. The decrease of bound water with increasing dextran molecular weight was attributed to the decrease in the number of free OH groups available for the interaction with water molecules owing to the formation of *intramolecular* hydrogen bonding between OH groups as the chains increased in length, or to steric hindrance between glucose units.^45^

Despite intensive interest in the hydration of macromolecules such as PEG and dextran and the experimental determination of hydration as mentioned above, the majority of techniques used for such measurements are suitable only for macromolecules in bulk solution. On the other hand, very few options are available to characterize the solvation capacity of polymer chains grafted onto a surface. In order to compare the solvation properties of PEG and dextran, and in particular those of PLL*-g*-dex and PLL*-g*-PEG, we investigated the hydration capacity of the two copolymers (i.e., the ability to incorporate aqueous buffer solution) by combining quartz crystal microbalance with dissipation monitoring (QCM-D) with optical waveguide lightmode spectroscopy (OWLS) measurements.^16^ The adsorbed mass sensed by QCM-D includes a contribution from the solvent molecules absorbed within the surface-bound polymer layer, in contrast to optical techniques, such as OWLS, which are sensitive only to the “dry mass” of polymer adsorbed onto the surface. By subtracting the “dry mass” derived from OWLS from the “wet mass” measured by QCM-D, it is therefore possible to calculate the mass of solvent absorbed per unit substrate area within the brush-like structure of surface-immobilized PLL*-g*-dex or PLL*-g*-PEG copolymers. This parameter is described as the areal solvation, Ψ, of the polymer brush.^16^

## Materials and Methods

### Poly(l-lysine)-*graft*-dextran (PLL*-g*-dex)

The synthesis of poly(l-lysine)-*graft*-dextran (PLL*-g*-dex) copolymers has been previously described in detail.^20,21^ Briefly, the molecules employed in this work were prepared by a reductive amination reaction of PLL·HBr (20 kDa, polydispersity 1.1, Sigma-Aldrich, Switzerland) with dextran (dextran T5, 5 kDa, T10, 10 kDa, and T20, 20 kDa, polydispersity 1.4–1.8, Pharmacosmos A/S, Denmark). Borate buffer (50 mM, pH 8.5) was used as the solvent for the reaction. An approximately 10× molar excess of sodium cyanoborohydride (NaBH_3_CN, Fluka Chemika, Switzerland) to dextran was used to reduce the unstable Schiff base resulting from the reaction between the terminal dextran aldehyde group and primary amino groups of PLL. The resulting copolymers were isolated by ultracentrifugation (Vivaspin 15R centrifugation tubes, 30000 MWCO, Sartorius AG, Switzerland) to remove the unreacted starting materials. Varying the ratio of Lys/dex allowed us to control the grafting ratio, *g*, defined as the number of lysine monomers per dextran chain.

PLL*-g*-dex molecules were characterized by ^1^H NMR spectroscopy and elemental analysis (EA). ^1^H NMR spectra of the copolymers in D_2_O were recorded on a Bruker spectrometer (300 MHz), and both NMR spectra and elemental-analysis data were used to evaluate the grafting ratio.

The notation PLL(*x*)-*g*[*y*]-dex(*z*) for the copolymers was used to represent the formula mass of PLL in kDa (*x*) (including the counterions, Br^–^, as a precursor), the formula mass of dextran in kDa (*z*), and the grafting ratio *g*[*y*] (defined as the number of lysine monomers/dextran side chain).

For comparison purposes, PLL(20)*-g*-PEG(5) copolymers (SuSoS AG, Dübendorf, Switzerland) have also been investigated, with PEG(5) side chains (polydispersity 1.03) attached to a PLL(20) backbone, covering a similar range of grafting ratios to that of the PLL*-g*-dex copolymers employed in a previous study.^17^

### Optical Waveguide Lightmode Spectroscopy (OWLS)

Optical waveguide lightmode spectroscopy (OWLS) was employed to determine the “dry mass” of polymer adsorbed onto the surface of a waveguide. Experiments were performed using an OWLS 110 instrument (Microvacuum, Budapest, Hungary).

OWLS is an optical biosensing technique for the *in situ*, label-free analysis of adsorption processes.^48^ The sensing principle of OWLS has been described in detail elsewhere;^48−50^ briefly, the adsorbed mass is calculated from the change in the refractive index in the vicinity of the waveguide surface upon adsorption of molecules. Since the solvent molecules coupled to the adsorbed polymer do not contribute to a *change* in the refractive index before and after the polymer adsorption, they also do not contribute to the adsorbed mass detected by OWLS (“dry mass”, i.e., dry areal mass density, *m*_dry_ [ng/cm^2^]).

The refractive index increment (d*n*/d*c*) of dextran was measured by means of a refractometer, and a value of 0.131 was used for all measurements to calculate the mass of polymer adsorbed. For all PLL*-g*-PEG copolymers, a value of 0.139 was used.^18^ Since the d*n*/d*c* values of dextran or PEG and PLL are very similar, no d*n*/d*c* correction was made for the different structures investigated.

Prior to the experiments, optical-waveguide chips (Microvacuum, Budapest, Hungary) consisting of a 1 mm-thick glass substrate and a 200 nm-thick Si_0.75_Ti_0.25_O_2_ waveguiding layer at the surface were ultrasonicated in 0.1 M HCl for 10 min, rinsed with Millipore water, ultrasonicated in 2-propanol for 10 min, rinsed again with Millipore water, and then dried under a dry nitrogen stream. The substrates were subsequently cleaned in a UV/ozone cleaner (Boeckel Industries Inc., Feasterville, PA, USA, model 135500) for 30 min.

The cleaned waveguides were assembled into the OWLS flow cell and equilibrated by exposure to a HEPES buffer solution (10 mM 4-(2-hydroxyethyl)piperazine-1-ethanesulfonic acid (Sigma, St. Louis, MO, USA), adjusted to pH 7.4 with 6.0 M NaOH solution) overnight in order to obtain a stable baseline. The waveguides were then exposed to a polymer solution (0.25 mg mL^–1^ in HEPES buffer) for at least 30 min, resulting in the formation of a polymer adlayer, and rinsed three times by soaking in a buffer solution for 30 min.

### Quartz-Crystal Microbalance (QCM)

All QCM-D measurements were performed with a commercial quartz-crystal microbalance with dissipation monitoring (Q-Sense E4, Gothenburg, Sweden). The instrument includes 4 sensors that were used in a parallel configuration.

QCM-D is sensitive to the viscoelastic properties and density of masses coupled to the mechanical oscillation of the quartz crystal. For the polymers employed in this work—PLL*-g*-dex and PLL*-g*-PEG—the measured adsorbed mass consists of the polymer along with solvent molecules that may be either hydrodynamically associated or strongly interacting (e.g., via hydrogen bonds) with the polymers. The total mass sensed by QCM-D, *m*_wet_, is therefore the mass of the polymer plus that of the absorbed solvent molecules.

The sensor crystals used in the measurements were 5-MHz AT-cut crystals sputter-coated with SiO_2_ (Q-Sense, Gothenburg, Sweden). The changes of resonance frequency (Δ*f*) and energy dissipation (Δ*D*) were measured simultaneously at 6 different overtones of the fundamental frequency (3rd overtone at 15 MHz, 5th overtone at 25 MHz, 7th overtone at 35 MHz, 9th overtone at 45 MHz, 11th overtone at 55 MHz, and 13th overtone at 65 MHz). Measurements at the fundamental frequency (5 MHz) were not considered due to this resonance being very sensitive to bulk-solution changes and generating unreliable data. The temperature of the QCM liquid chambers was stabilized at 25 ± 0.02 °C.

All measurements were performed under flow conditions. The resonance frequency, *f*_0_, and the dissipation factor, *D*, of the quartz crystals were measured first in aqueous HEPES buffer solution in order to set the baseline. The polymer-free aqueous HEPES buffer solution was then replaced with a PLL*-g*-dex- or PLL*-g*-PEG-containing aqueous HEPES buffer solution (0.25 mg/mL). After adsorption for 30 min (at a flow rate of 20 μL/min), the liquid cell was rinsed with polymer-free aqueous HEPES buffer solution to confirm that no noticeable polymer desorption had occurred.

The areal solvation of the brush copolymers, Ψ, defined as the mass of solvent molecules per unit area absorbed within the structure of the surface-bound copolymers, can be derived by subtracting the “dry mass”, *m*_dry_, calculated from OWLS measurements, from the “wet mass”, *m*_wet_, calculated from QCM-D measurements. In the present work, *m*_wet_ was calculated using the Sauerbrey equation^51^
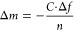
1where *Δm* is the change in the total mass of the crystal induced by adsorption, *Δf* is the change in frequency, *n* is the overtone number, and *C* is a constant that is characteristic of the crystal (*C* = 17.7 ng/Hz for a 5 MHz quartz crystal).

## Results and Discussion

### Synthesis and Structural Features of PLL*-g*-dex Copolymers

PLL*-g*-dex copolymers with different dextran molecular weights and grafting ratios were synthesized. The nominal molecular weights of the selected dextran were 5, 10, and 20 kDa (denoted as dex(5), dex(10), and dex(20)). The grafting ratio was varied between roughly 3 and 9 and evaluated by means of NMR and elemental analysis. As a reference, a series of PLL(20)*-g*-PEG(5) (20 kDa for the nominal molecular weight of the PLL backbone and 5 kDa for the nominal molecular weight of PEG side chains) with varying grafting ratios, roughly *g*[3] to *g*[11], was purchased from SuSoS AG (Dübendorf, Switzerland). The actual molecular weights of dextran, PEG, and PLL used for the synthesis of the copolymers are reported in Table 1. The three dextran polymers with different molecular weights, dex(5), dex(10), and dex(20), were selected to maintain the structural features that may critically influence the adsorption properties and be comparable with those of PLL(20)*-g*-PEG(5); dex(5) is nearly identical with PEG(5) in molecular weight, PLL*-g*-dex(10) can potentially generate comparable film thicknesses with those of PLL*-g*-PEG(5) copolymers as dex(10) has a comparable fully extended chain length to that of PEG(5) (40.5 nm for PEG(5) and 44.6 nm for dex(10), respectively),^21^ and dex(20) is composed of a similar number of monomer units to PEG(5) (126.5 sugar rings for dex(20) and 109.9 EG monomers for PEG(5)). The detailed structural features of all of the copolymers employed in this study are shown in Table 1.

**Table 1:** Synthesized PLL*-g*-dex Copolymers and PLL*-g*-PEG Copolymers, Used for Comparison Purposes^a^

Polymer	Synthesis yield [%]	No. of grafted side chains per PLL	No. of free lysines per PLL	Percentage of side-chain grafting (%)	M.W. of copolymer (kDa)
*PLL(20)-*g*[3.4]-dex(5)*	30	37.0	88.8	29.4	207
*PLL(20)-*g*[5.3]-dex(5)*	60	23.7	102.1	18.9	139
*PLL(20)-*g*[7.3]-dex(5)*	27	17.2	108.6	13.7	105
*PLL(20)-*g*[8.7]-dex(5)*	60	14.5	111.4	11.5	91
*PLL(20)-*g*[3.7]-dex(10)*	46	34.0	91.8	27.0	356
*PLL(20)-*g*[4.8]-dex(10)*	40	26.2	99.6	20.8	278
*PLL(20)-*g*[6.5]-dex(10)*	59	19.4	106.5	15.4	210
*PLL(20)-*g*[8.6]-dex(10)*	58	14.6	111.2	11.6	162
*PLL(20)-*g*[1.7]-dex(20)*	36	76.3	49.6	60.6	1580
*PLL(20)-*g*[3.6]-dex(20)*	70	35	90.9	27.8	733
*PLL(20)-*g*[5.1]-dex(20)*	53	24.6	101.3	19.5	520
*PLL(20)-*g*[8.8]-dex(20)*	69	14.4	111.5	11.4	284
*PLL(20)-*g*[3]-PEG(5)*	-	40.7	81.3	33.3	212
*PLL(20)-*g*[4.4]-PEG(5)*	-	27.8	94.3	22.7	150
*PLL(20)-*g*[6.6]-PEG(5)*	-	18.5	103.5	15.2	105
*PLL(20)-*g*[11.2]-PEG(5)*	-	10.3	104.6	8.9	64

a Actual molecular weights of dex, PEG, and PLL: dex(5), 5.157 kDa; dex(10), 10 kDa; dex(20), 20.5 kDa; PEG(5), 4.834 kDa; PLL(20), 26.3 kDa for all PLL-*g*-dex copolymers, except PLL(20)-*g*[3.6]-dex(20); 25.5 kDa for all PLL-*g*-PEG copolymers, except PLL(20)-*g*[11.2]-PEG(5); 24 kDa for PLL(20)-*g*[3.6]-dex(20) and PLL(20)-*g*[11.2]-PEG(5).

### Comparative Adsorption Measurements Using OWLS and QCM-D

The areal solvation, Ψ, of the surface-bound, brush-like copolymers employed in this work was determined by applying the combined experimental approach of OWLS and QCM-D. *In-situ* OWLS measurements were performed to obtain the average “dry mass” (*m*_dry_) of the surface-adsorbed copolymers. From the adsorbed masses and the compositional features of the copolymers, it was possible to calculate the surface density of lysine monomer, *n*_lys_, and dextran or PEG chains, *n*_dex or PEG_, the former reflecting the number of copolymer units on the surface and the latter reflecting the efficacy of the copolymer in grafting the hydrophilic polymer chains (dextran or PEG) onto the surface. QCM-D was used to quantify the “wet mass” (*m*_wet_), consisting of the mass of polymer along with solvent molecules adsorbed within the brush structure. Ψ values were calculated by subtracting the “dry mass”, *m*_dry_, obtained by OWLS, from the “wet mass”, *m*_wet_, obtained by QCM-D. The adsorption data determined by OWLS and QCM-D are reported in Table 2 and Table 3, respectively.

**Table 2:** Summary of the Adsorption Data Determined by OWLS for the PLL*-g*-dex and PLL-g-PEG Copolymers Investigated (*m*_dry_ = Adsorbed Polymer Mass Determined by OWLS, *n*_lys_ = Surface Density of Lysine Monomers, *n*_dex or PEG_ = Surface Density of Dextran or PEG, *n*_monomer units dex or PEG_ = Surface Density of the Monomer Units of Dextran or PEG, and *L*/2*R*_g_ = Extent of Overlap between PEG or Dex Chains on the Surface Where *L* = Distance between PEG or Dex Chains, *R*_g_ = Radius of Gyration)^a^

Surface	*m*_dry_ [ng/cm^2^]	*n*_lys_ [1/nm^2^]	*n*_dex or PEG_ [1/nm^2^]	*n*_monomer units dex or PEG_ [1/nm^2^]	*L*/2*R*_g_
*PLL(20)-g[3.4]-dex(5)*	244 ± 44	0.89 ± 0.20	0.26 ± 0.05	8.36 ± 1.51	0.43
*PLL(20)-g[5.3]-dex(5)*	290 ± 16	1.59 ± 0.09	0.30 ± 0.02	9.53 ± 0.52	0.40
*PLL(20)-g[7.3]-dex(5)*	269 ± 57	1.94 ± 0.41	0.27 ± 0.06	8.45 ± 1.80	0.42
*PLL(20)-g[8.7]-dex(5)*	190 ± 21	1.59 ± 0.17	0.18 ± 0.02	5.80 ± 0.62	0.51
*PLL(20)-g[3.7]-dex(10)*	319 ± 28	0.68 ± 0.06	0.18 ± 0.02	11.31 ± 0.99	0.36
*PLL(20)-g[4.8]-dex(10)*	290 ± 17	0.79 ± 0.05	0.16 ± 0.01	10.16 ± 0.58	0.38
*PLL(20)-g[6.5]-dex(10)*	397 ± 71	1.44 ± 0.26	0.22 ± 0.04	13.63 ± 2.43	0.33
*PLL(20)-g[8.6]-dex(10)*	321 ± 2	1.50 ± 0.01	0.17 ± 0.00	10.75 ± 0.07	0.37
*PLL(20)-g[1.7]-dex(20)*	347 ± 83	0.17 ± 0.04	0.10 ± 0.02	12.77 ± 3.03	0.34
*PLL(20)-g[3.6]-dex(20)*	313	0.33	0.09	11.37	0.36
*PLL(20)-g[5.1]-dex(20)*	345 ± 29	0.52 ± 0.04	0.10 ± 0.01	12.43 ± 1.05	0.34
*PLL(20)-g[8.8]-dex(20)*	397	0.99	0.11	13.98	0.33
*PLL(20)-g(3)-PEG(5)*	160 ± 3	0.54 ± 0.00	0.19 ± 0.00	20.49 ± 0.07	0.44
*PLL(20)-g[4.4]-PEG(5)*	176 ± 8	0.84 ± 0.04	0.20 ± 0.01	21.69 ± 1.07	0.43
*PLL(20)-g[6.6]-PEG(5)*	207 ± 45	1.41 ± 0.30	0.22 ± 0.05	24.24 ± 5.20	0.41
*PLL(20)-g[11.2]-PEG(5)*	174 ± 22	1.82 ± 0.22	0.17 ± 0.02	18.51 ± 2.26	0.46

a The error bars originate from the standard deviation of the mean values over repeated measurements.

**Table 3:** Summary of the Adsorption Data Determined by QCM-D for the PLL*-g*-dex and PLL-*g*-PEG Copolymers Investigated (*m*_wet_ = Adsorbed Polymer Mass Determined by QCM-D, Ψ = Areal Solvation, *m*_wet_ – *m*_dry_, *n*_H_2_O/HG_ = Number of Water Molecules per Hydrophilic Group)

Surface	*m*_wet_ [ng/cm^2^]	Ψ [ng/cm^2^]	*n*_H_2_O/HG_
*PLL(20)-g[3.4]-dex(5)*	929	686	8.0
*PLL(20)-g[5.3]-dex(5)*	749	459	6.2
*PLL(20)-g[7.3]-dex(5)*	710	442	5.2
*PLL(20)-g[8.7]-dex(5)*	611	421	6.7
*PLL(20)-g[3.7]-dex(10)*	1085 ± 66	766 ± 94	5.6 ± 3.1
*PLL(20)-g[4.8]-dex(10)*	961	671	5.2
*PLL(20)-g[6.5]-dex(10)*	1002 ± 36	605 ± 107	3.3 ± 1.1
*PLL(20)-g[8.6]-dex(10)*	920	599	4.0
*PLL(20)-g[1.7]-dex(20)*	1331	985	5.2
*PLL(20)-g[3.6]-dex(20)*	1411 ± 13	1098	4.9
*PLL(20)-g[5.1]-dex(20)*	1214	869	3.2
*PLL(20)-g[8.8]-dex(20)*	1271	874	3.9
*PLL(20)-g(3)-PEG(5)*	1115	992	20.6
*PLL(20)-g[4.4]-PEG(5)*	1039	863	14.9
*PLL(20)-g[6.6]-PEG(5)*	821 ± 54	614 ± 99	8.4 ± 0.6
*PLL(20)-g[11.2]-PEG(5)*	674 ± 25	500 ± 47	7.8 ± 0.4

Both OWLS and QCM-D revealed the rapid adsorption of all copolymers investigated onto the substrates. Upon exposure of the surface to the polymer solution, more than 90% of the final adsorbed mass was reached within the first 5 min, and no apparent polymer desorption could be observed upon rinsing with buffer solution.

### Comparison of the Solvation Capabilities of PLL(20)-*g*-PEG(5) and PLL(20)-*g*-dex(10) Copolymers

#### Areal Solvation (Ψ) as a Function of *g*

**Figure 2:**
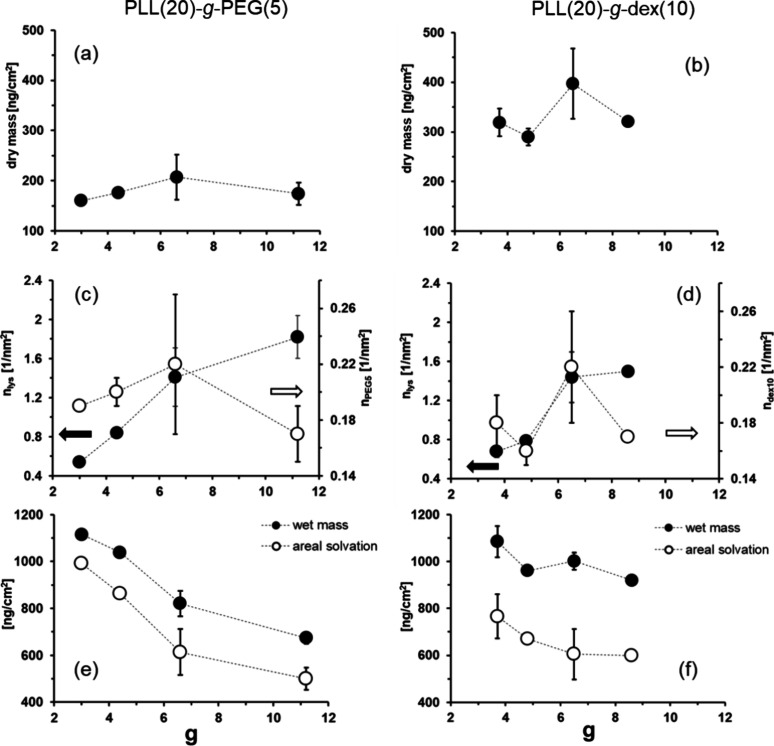
Plots of (a) dry mass of PLL(20)-*g*-PEG(5), (b) dry mass of PLL(20)-*g*-dex(10), (c) surface density of lysine and PEG(5) chains for PLL(20)-*g*-PEG(5), (d) surface density of lysine and surface density of dex(10) chains for PLL(20)-*g*-dex(10), (e) wet mass and areal solvation (ψ) for PLL(20)-*g*-PEG(5), and (f) wet mass and areal solvation (ψ) for PLL(20)-*g*-dex(10).

The dry mass, surface density of lysine monomers, *n*_lys_, surface density of hydrophilic polymer chains, *n*_dex_ or *n*_PEG_, wet mass, *m*_wet_, and areal solvation,Ψ, for PLL(20)-*g*-PEG(5) and PLL(20)-*g*-dex(10) copolymers are presented as a function of grafting ratio, *g*, in Figure 2 (the results for PLL(20)-*g*-dex(5) and PLL(20)-*g*-dex(20) are presented in Figure S1 in the Supporting Information).

For both PLL(20)-*g*-PEG(5) and PLL(20)-*g*-dex(10), the average values of dry mass showed a local maximum at *g* = 6.6 for PLL(20)-*g*-PEG(5) (Figure 2(a)) and *g* = 6.5 for PLL(20)-*g*-dex(10) (Figure 2(b)). This behavior can be readily understood by taking into account the changes of (i) the surface density of lysine monomers, *n*_lys_ (Figure 2(c) and 2(d)), which is gradually decreasing with decreasing *g*, and (ii) the molecular weights of the copolymers (Table 1), which are gradually increasing with decreasing *g*. On a PLL backbone, increasing grafting of lysine monomers with either PEG or dex chains tends to retard facile adsorption of the copolymers onto negatively charged surfaces due to the decrease in available anchoring units (i.e., free NH_2_/NH_3_^+^ units) as well as steric repulsion between neighboring PEG or dex side chains,^18,52^ unless the overlap between the side chains is minimal.^18^ Thus, the dry mass, i.e., the collective mass of all of the surface-adsorbed copolymers, is a compromise between these two opposing trends as a function of *g*. Similarly, the surface density of PEG(5) or dex(10) chain density on the surface, i.e., *n*_PEG5_ or *n*_dex10_, showed a local maximum as a function of *g* (Figure 2(c) and 2(d)). As an example, the *n*_lys_ of PLL(20)-*g*[3]-PEG(5), which reflects the surface density of the copolymer as well, is only ca. 30% that of PLL(20)-*g*[11.2]-PEG(5); however, the grafting density of PEG chains on a PLL backbone of PLL(20)-*g*[3]-PEG(5) is ca. 370% that of PLL(20)-*g*[11.2]-PEG(5). Thus, the *n*_PEG_ of PLL(20)-*g*[3]-PEG(5) (0.19 ± 0.00) turned out to be very similar to that of PLL(20)-*g*[11.2]-PEG(5) (0.17 ± 0.02) (Table 2).

**Figure 3:**
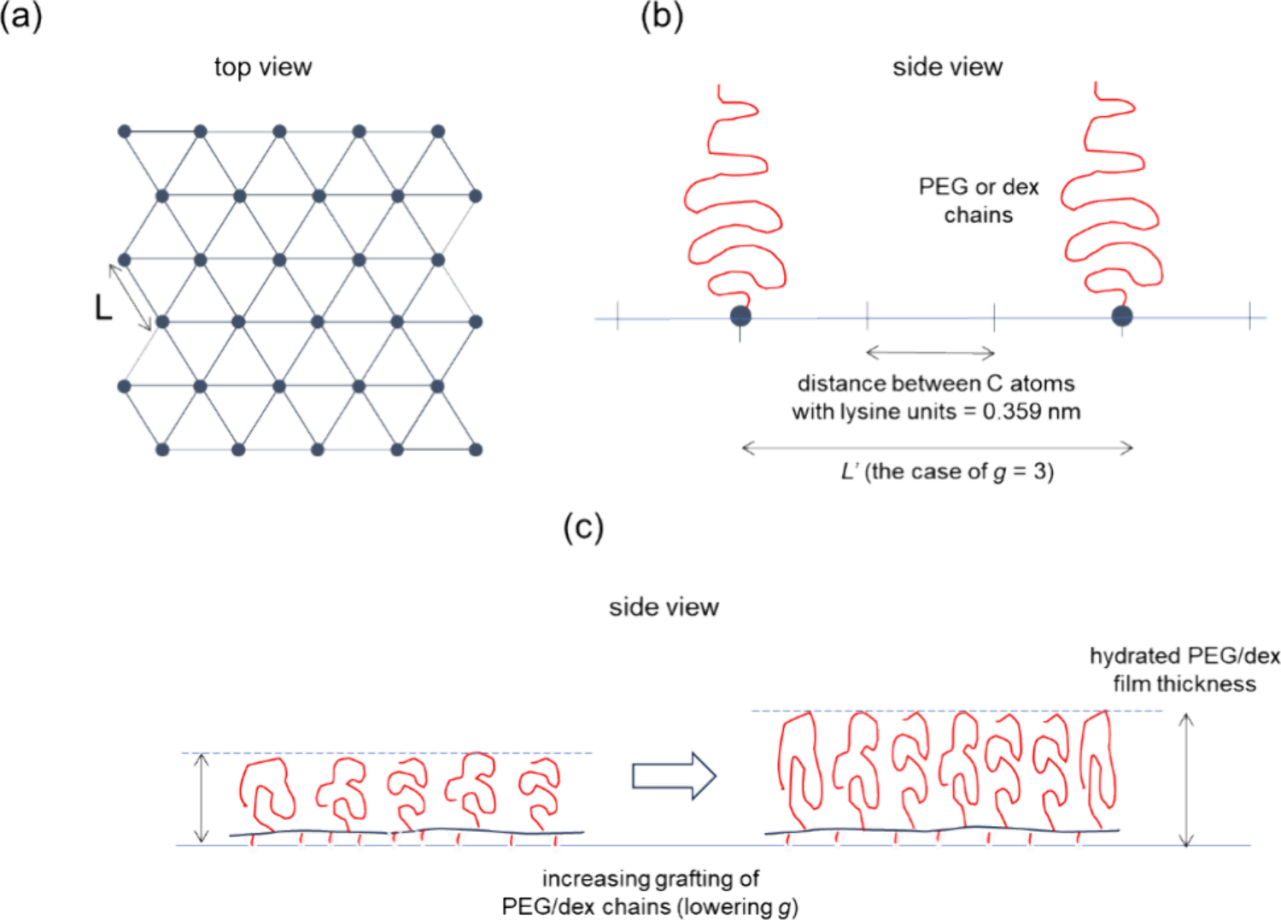
Schematic illustrations of (a) arrangement of PEG or dex chains anchoring sites in a 2-dimensional hexagonal pattern^18^ to estimate the *average* distance (*L*) between the side chains (top view) (each dot represents the location of grafted PEG or dex chains) and (b) arrangement of PEG or dex chains anchoring sites along a PLL backbone (side view) in a linear fashion to estimate the average local distance (*L*′) between the side chains (side view). Shown in the schematic is the case of *g* = 3 (c) increasing hydrated PEG or dex film thickness, and thus increasing areal solvation (ψ), with decreasing *g*, following the stretching of side chains to relieve the increasing lateral steric repulsion.

The wet masses of both series were ca. 2.5–7 times larger than the corresponding dry masses and thus are dominated by the changes in areal solvation, Ψ, when varying *g*. It is interesting that, despite the decreasing trend of dry mass and surface density of hydrophilic chains when *g* ≤ 6.5 or 6.6, the wet mass and areal solvation continue to increase with decreasing *g* (Figure 2(e) and 2(f)). This means that the thickness of hydrated layers of PLL-*g*-PEG or PLL-*g*-dex, composed of predominantly hydrated PEG or dex chains (assuming that PLL backbone chains are lying flat on the surface), continues to increase even though the density of PEG or dex chains is slightly decreasing with decreasing *g* when *g* ≤ 6.5 or 6.6. To understand this behavior, it must be first considered that the surface densities of PEG or dex chains in Table 2 are the estimated *average* values by assuming that individual hydrophilic chains are equally spaced, in a 2-dimensional hexagonal arrangement (see Figure 3(a) for a graphic illustration).^18^ In reality, however, as PEG or dex chains are grafted onto PLL backbones and then adsorb onto oxide surfaces via the free NH_2_/NH_3_^+^ units on the PLL backbones, the PEG or dex belonging to the same PLL backbones tend to cluster together. With decreasing *g* values, i.e., as the number of grafted PEG/dex chains along a PLL backbone increases, the average PEG/dex chain density along the backbone, namely, the *local* chain density, can be much higher than the *average* chain density on the surface after adsorption.

**Figure 4:**
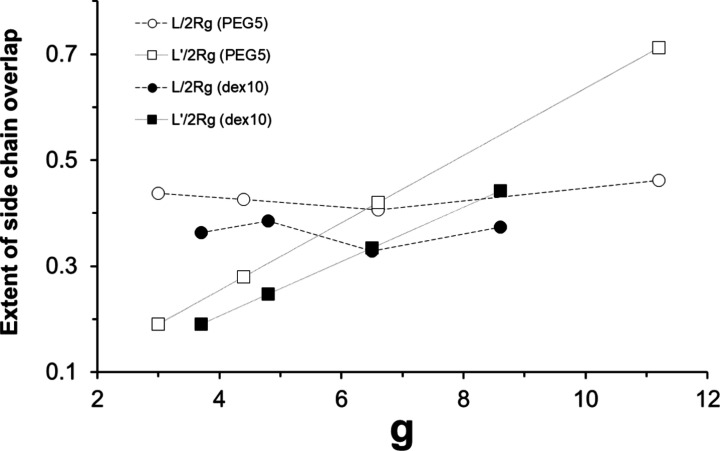
Extent of side chain overlap between PEG(5) chains or between dex(10) chains on the surface, as estimated from *L*/2*R*_g_ or *L*′/2*R*_g_, where *L* is the measured average distance between polymer chains on the surface, assuming a 2-dimensional hexagonal arrangement (Figure 3(a)), *L*′ is the average local distance between polymer chains along a fully extended PLL chain (Figure 3(b)), and *R*_g_ is the radius of gyration of each polymer chain.

In order to discuss this behavior in a quantitative manner, the distance between PEG or dex side chains on a single PLL chain is estimated as follows: assuming that only one PLL-*g*-PEG or PLL-*g*-dex molecule is allowed to adsorb onto the surface with a fully stretched PLL backbone^52^ (see Figure 3(b)) (please note that all the bond angles between the atoms of PLL are assumed to be preserved in thermodynamic equilibrium; see Figure S2), the average distance between the carbon atoms with grafted side chains, defined as *L*′, is 0.359 nm × *g* (the detailed calculation of the spacing between the carbon atoms with the grafted side chains, i.e., 0.359 nm, is shown in the Supporting Information, Figure S2). This arrangement is, of course, hypothetical to estimate an upper bound for the distance between neighboring PEG or dex side chains on a PLL backbone adsorbed on the surface. The extent of overlap between neighboring PEG or dex chains can be then estimated by calculating *L*′/2*R*_g_. *R*_g_ is the radius of gyration and is reported to be 3.49 nm for dex(10) chains^21^ and 2.82 nm for PEG(5).^18^ The results are shown in Figure 4 and compared with *L*/2*R*_g_ values for PLL(10)-*g*-PEG(5) and PLL(10)-*g*-dex(10) addressed in Table 2. It should be noted that *L*/2*R*_g_ or *L*′/2*R*_g_ is inversely proportional to the chain overlap. A table for the distances between PEG or dex chains along the same PLL backbone, *L*, and the ratio between *L*/*L*′ (Table S1), as well as the plots of *L* or *L*′ vs *g* (Figure S3), are presented in the Supporting Information.

Comparing the PLL(20)-*g*-dex(10) and PLL(20)-*g*-PEG(5) copolymers, both *L*/2*R*_g_ and *L*′/2*R*_g_ values are smaller for the dex(10) version, which means that dex(10) chains are more overlapped on average, both on the surface (*L*/2*R*_g_) and along a single PLL backbone (*L*′/2*R*_g_). This is mainly due to the bulkier size (*R*_g_) of dex(10) than PEG(5). Next, for both PLL(20)-*g*-PEG(5) and PLL(20)-*g*-dex(10) copolymers, the changes in *L*/2*R*_g_ values are very small within the range of varied *g*, whereas *L*′/2*R*_g_ values change more drastically as a function of *g*. Nevertheless, a magnified plot of *L*/2*R*_g_ values for PLL(20)-*g*-PEG(5) alone showed a similar trend with those of dry mass (Figure 2(a)) and *n*_lys_ (Figure 2(c)), where the highest overlap is observed from *g* = 6.5. Meanwhile, the corresponding plot of *L*/2*R*_g_ values for PLL(20)-*g*-dex(10) shows fluctuating values within the varied *g*, also similarly to Figure 2(b) and Figure 2(d); the plots are presented in the Supporting Information, Figure 4S. Most importantly, for both PLL(20)-*g*-PEG(5) and PLL(20)-*g*-dex(10), the relative magnitude of *L*/2*R*_g_ and *L*′/2*R*_g_ as a function of *g* starts to be reversed around *g* = 6.5, such that *L*′/2*R*_g_ > *L*/2*R*_g_ when *g* > 6.5 and *L*′/2*R*_g_ > *L*/2*R*_g_ when *g* < 6.5. Thus, when *g* > 6.5, the PEG/dex chains belonging to the same PLL backbones or different PLL backbones nestle together to yield the average spacing, *L*, and the extent of packing, *L*/2*R*_g_, on the surface. This can be most readily achieved by the adsorption of a high density of copolymers onto the surface; the highest surface density of lysine monomers, *n*_lys_, by PLL(20)-*g*[11.2]-PEG(5) and PLL(20)-*g*[8.6]-dex(10) for each series of copolymers (Table 2) is consistent with this. In contrast, when *g* < 6.5, the extent of packing along the PLL backbone, *L*′/2*R*_g_, is clearly higher than the average values on the surface; for example, the *L*/*L*′ ratio for PLL(20)-*g*[3.0]-PEG(5) is 2.29. In bulk solution, the steric repulsion between neighboring side chains of a single copolymer molecule can be relieved by arranging the side chains radially along the PLL backbone.^52^ When a single PLL-*g*-PEG or PLL-*g*-dex molecule is adsorbed onto the surface, the volume over which the side chains can be distributed to relieve steric repulsion is reduced by half. Finally, when many PLL-*g*-PEG or PLL-*g*-dex molecules are allowed to adsorb onto the surface and reach equilibrium, the volume for distributing neighboring side chains to relieve steric repulsion becomes even more restricted. At this point, the only remaining degree of freedom for relieving the steric repulsion is to stretch the side chains, especially the portion of side chains near to the backbone, toward the bulk solution. This leads to an increase in the hydrated film thickness of PEG or dex chains with decreasing *g*. This scenario is graphically presented in Figure 3(c) and can possibly explain the continuous increase in wet mass and areal solvation of PLL(20)-*g*-PEG(5) and PLL(20)-*g*-dex(10) (Figures 2(e) and (f)), despite the gradual decrease in dry mass and hydrophilic chain density when *g* ≤ 6.5 or 6.6 (Figures 2(c) and (d)).

It is also important to note that, even though this trend is common for both copolymers, the extent of increase is clearly higher for PLL(20)-*g*-PEG(5) than for PLL(20)-*g*-dex(10) (Figure 2(e) and (f)); when the range between *g* = 6.6 (PEG(5)) or 6.5 (dex(10)) to *g* = 3 (PEG(5)) or 3.7 (dex(10)) is taken into account, Δψ/Δ*g* = 110.5 for PLL(20)-*g*-PEG(5) and 57.5 for PLL(20)-*g*-dex(10). If the increasing trend of the wet mass and areal solvation with decreasing *g* is due to the stretch of PEG or dex chains with decreasing *g* as addressed above, the smaller Δψ/Δ*g* for PLL(20)-*g*-dex(10) is attributed to the relatively stiffer characteristics of dex chains compared to PEG.^22−24,45^ Many past studies have agreed that dextran chains tend to retain relatively stiff structures whether they are present in bulk solution^45^ or grafted onto the surface.^22−24^

#### Number of Water Molecules per Monomer Units (*n*_H_2_O/mon unit_) vs Hydrophilic Groups (*n*_HG_)

**Figure 5:**
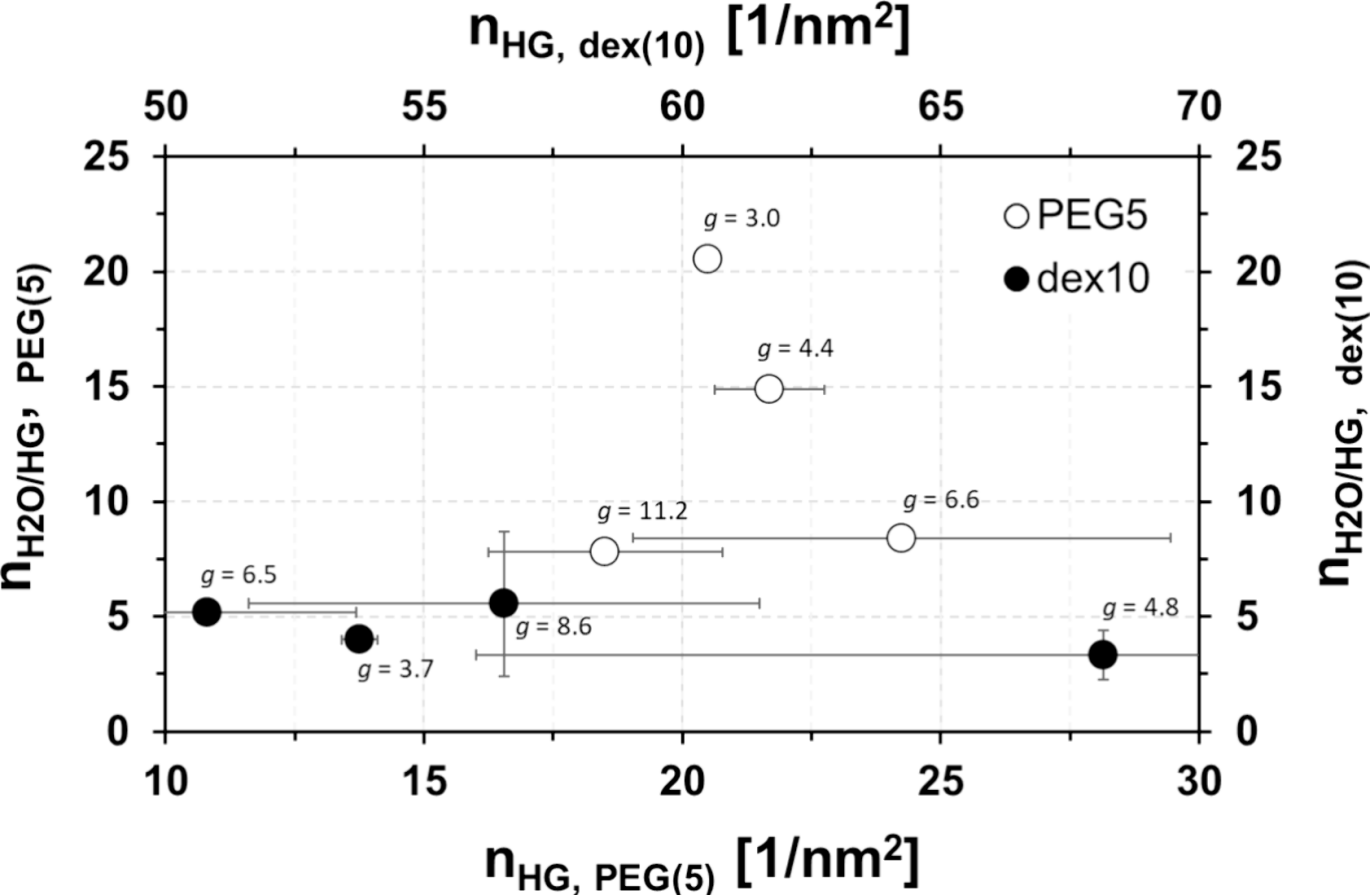
Number of water molecules per hydrophilic group, *n*_H_2_O/HG_, as a function of the surface density of hydrophilic groups (*n*_HG_) for PLL(20)-*g*-PEG(5) and PLL(20)-*g*-dex(10) copolymers.

In order to more clearly visualize the different capabilities of PLL(20)-*g*-PEG(5) and PLL(20)-*g*-dex(10) copolymers to incorporate water, the number of water molecules absorbed per monomer unit (EG monomer or sugar ring), *n*_H_2_O/mon unit_, was determined for each copolymer by applying the following equations^16^
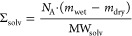
2
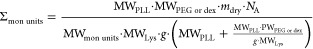
3
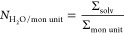
4where Σ_solv_ and Σ_mon units_ are the areal densities of the solvent molecules and monomer units, respectively, *N*_A_ is the Avogadro constant, MW_solv_ is the molecular weight of the solvent, MW_PLL_ is the molecular weight of the PLL backbone, MW_Lys_ is the molecular weight of a lysine monomer, MW_PEG or dex_ and MW_mon unit_ are the molecular weights of the hydrophilic side chains, PEG or dextran, and of the monomer unit (EG monomer or dextran ring), respectively, and *g* is the grafting ratio. From *n*_H_2_O/mon unit_, from the surface density of dextran or PEG chains determined by OWLS (*n*_dex or PEG_), and from the compositional features of the copolymers, it is then possible to calculate the number of water molecules per hydrophilic group (HG), *n*_H_2_O/HG_, having considered in this work one hydrophilic group (the ether oxygen, −O−) per EG monomer and five hydrophilic groups (3 −OH and 2 −O−) per sugar unit. The resulting values are presented in Figure 5 as a function of the surface density of hydrophilic groups, *n*_HG_.

It is worth commenting on three features. First, the number of water molecules surrounding the hydrophilic groups for both PLL(20)-*g*-PEG(5) and PLL(20)-*g*-dex(10) series, as detected by the QCM-D/OWLS approach, is generally higher than that acquired via other methods, such as DSC^53^ or the acoustic method;^45^ for example, a minimum of 2–3 water molecules forming hydrogen bonds with one EG monomer is needed to satisfy the basic hydration requirements of PEG,^54,55^ in the case of PLL-*g*-PEG copolymers. Indeed, about 3 water molecules were detected for each EG monomer unit by DSC,^53^ while the present study detected 5–6 to 13–14 *additional* water molecules per ethylene oxide unit, depending on the grafting ratio. In the case of dextran, a DSC study by Gekko et al. showed that the amount of bound water molecules per OH group stays constant at about 0.5 for molecular weights of dextran between 2 and 50 kDa, whereas the present study detected 2.5–4.5 *more* water molecules per −OH unit. The reason for the higher number of water molecules for both PEG and dextran chains can be first ascribed to the employment of surface-grafted polymers in the present study, some of which contain highly stretched chains. Previous studies focused on polymer chains in bulk solution. In other words, the monomer density is higher when polymer chains are grafted onto the surface than in bulk solution, and consequently, the total amount of associated water molecules is also higher. In addition, the higher number of water molecules for both PEG and dextran chains could be partly due to the dynamic nature of QCM-D, which may detect a higher number of water molecules than a static method such as DSC. Second, in comparison between PLL(20)-*g*-PEG(5) and PLL(20)-*g*-dex(10), the number of water molecules for each hydrophilic group is clearly higher for PLL(20)-*g*-PEG(5). Although *n*_HG_ values are higher for PLL(20)-*g*-dex(10) copolymers (ca. 51–68 for PLL(20)-*g*-dex(10) vs ca. 19–24 for PLL(20)-*g*-PEG(5) on average), the highest *n*_H_2_O/HG_ value revealed by PLL(20)-*g*-dex(10) (5.6 water molecules on average per hydrophilic group for the case of PLL(20)-*g*[3.7]-dex(10)) is about a quarter of the highest *n*_H_2_O/HG_ value shown by PLL-*g*-PEG copolymers (20.6 water molecules on average per hydrophilic group for the case of PLL-*g*[3]-PEG) and even lower than the lowest value relative to the PLL-*g*[11.2]-PEG (7.8 water molecules on average per hydrophilic group). Even when only OH groups are taken into account as hydrophilic groups, the values of *n*_H_2_O/HG_ for all of the copolymers employed remain significantly lower than those of the PLL-*g*-PEG copolymers (data not shown). This superior hydrating capability of EG with respect to glucose units may be closely associated with the presence of ordered EG–water complexes. However, the QCM-D/OWLS approach cannot distinguish “tightly bound” vs “loosely bound” water molecules, either for PEG (EG) or dextran (glucose), and other parameters such as flexibility of polymer chains may play a role too. Further studies to selectively measure water molecules with different extents of association would be very helpful in clarifying this issue in the future. Third, the *n*_H_2_O/HG_ values of PLL(20)-*g*-PEG(5) are clearly increasing with decreasing *g* (and thus the highest *n*_H_2_O/HG_ value is observed from *g* = 3.0), and this trend has the same physicochemical origin with the areal solvation addressed above. In contrast, the *n*_H_2_O/HG_ values of PLL(20)-*g*-dex(10) remain virtually unaffected by the variation of *g*. This behavior also has the same origin as the lower sensitivity of areal solvation to changes *g* for PLL(20)-*g*-dex(10) compared to PLL(20)-*g*-PEG(5) (Figure 2(e) and (f)), i.e., the stiffer structure of dex chains.

### Comparison of the Solvation Capabilities of PLL(20)-*g*-dex Copolymers with Different Molecular Weights of Dextran

Previously, the relatively stiff configuration of dextran chains (Figure 1b) and its influence on hydration were reported mainly for the polymers in bulk solution with varying molecular weights. For example, Gekko et al.^45^ reported that the amount of hydration per OH group starts to increase with decreasing molecular weight when Mn < 2 kDa, whereas it stays constant when Mn > 2 kDa. This was attributed to the conformational change of dextran chains from random coil to stretch below a threshold of ca. 2 kDa. In other words, the accessibility of dextran chains by water molecules is more enhanced at lower chain lengths, due to the stiffness of the glycan chains. The condition is somewhat different in the present study, as all the dextran chains are grafted onto a surface, and even though the molecular weights of the dextran chains (5–20 kDa) are well above the threshold reported in Gekko’s study (2 kDa), all of them display highly stretched conformations according to the calculations of *L*/2*R*_g_ (0.33–0.51, Table 2). Thus, a focus of this study is to explore how the hydration capacity changes for dextran chains compared to PEG chains when both chains are comparably highly stretched.

**Figure 6:**
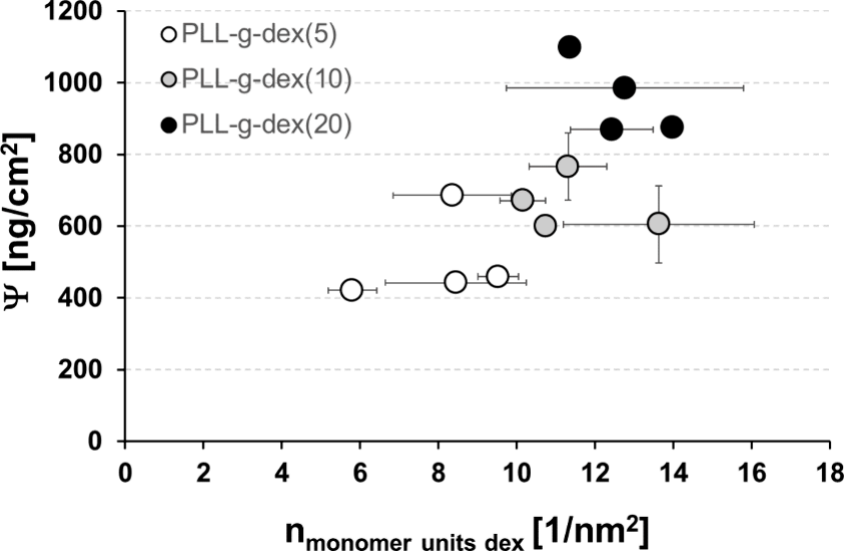
Areal solvation, Ψ, as a function of the surface density of sugar units (*n*_monomer units dex_) for adsorbed PLL(20)-*g*-dex copolymers differing in both the molecular weight of the dextran side chains and the grafting ratio.

To this end, the analysis of solvation for three series of copolymers was carried out to reveal the relationship between hydration capabilities and the number of monomer units or the number of hydrophilic groups. First, the values of areal solvation (ψ) for all PLL-*g*-dex copolymers investigated are plotted against the surface density of monomer units, *n*_monomer units dex_, in Figure 6.

**Figure 7:**
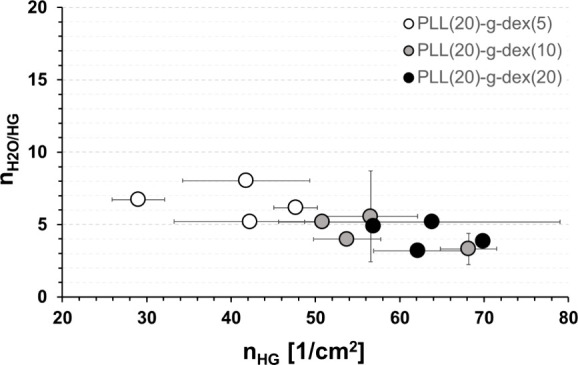
Number of water molecules per hydrophilic group, *n*_H_2_O/HG_, as a function of the surface density of hydrophilic groups (*n*_HG_) for PLL(20)-*g*-dex copolymers differing in both the molecular weight of the dextran side chains and the grafting ratio.

As shown in Figure 6, the Ψ values for the three series of copolymers clearly increased in the order PLL-*g*-dex(5) < PLL-*g*-dex(10) < PLL-*g*-dex(20). This is obviously due to the increasing number of monomer units, i.e., glycan rings, on the surface in that order. In turn, the number of water molecules per hydrophilic group, *n*_H_2_O/HG_, is plotted against the surface density of hydrophilic groups, *n*_HG_, in Figure 7, which shows whether the number of water molecules for each hydrophilic group, i.e., −OH or −O– of the glycans, changes with varying molecular weight, as well as with the degree of extension on the surface.

In fact, this is an extension of Figure 5, which was for the series of PLL(20)-*g*-dex(10), to the other two series of PLL-*g*-dex. Although *n*_H_2_O/HG_ values appear to decrease with increasing *n*_HG_ overall, this trend is very weak, especially considering the overlapped error bars. In other words, within the given range, the variation of *n*_HG_ does not significantly alter the number of water molecules per hydrophilic group within dextran chains. This is in stark contrast to the behavior of the PLL(20)-*g*-PEG(5) series (Figure 5). Again, this behavior can be explained by the observation that, unlike PEG chains, hydrophilic groups of dextran chains are only randomly and weakly hydrated by surrounding water molecules.

### Implications of Hydration Behavior for Lubricating and Antifouling Properties

The differences in the hydration behavior of the PLL-*g*-PEG and PLL-*g*-dex copolymers might be related to their different lubricating performance. A previous study comparing the boundary-lubricating properties of the PLL(20)-*g*-PEG(5) and PLL(20)-*g*-dex(10) copolymers employed in the present study^21^ revealed a slight but noticeable improvement in lubricating efficacy for PLL(20)-*g*-PEG(5) copolymers compared to PLL(20)-*g*-dex(10) in the low-speed regime (≤5 mm s^–1^), regardless of the grafting ratio, i.e., not only between the two copolymers with comparable *g* values, but also all PLL(20)-*g*-PEG(5) copolymers compared to all PLL(20)-*g*-dex(10) copolymers in Table 2. Thus, even if the coefficients of friction are plotted against the density of PEG or dextran chains, *n*_PEG or dex_, as in this study, the lubricating properties of PLL(20)-*g*-PEG(5) are still superior to those of PLL(20)-*g*-dex(10). It is noted that the clearly superior lubricity of PLL(20)-*g*-PEG(5) compared to PLL(20)-*g*-dex(10) was observed only within the low-speed regime (≤5 mm s^–1^), whereas the relative lubricating capabilities between the two copolymers were more complicated at higher speeds.^21^ This is because the lubricating properties of the surface grafted polymer chains are most directly governed by the chain conformation in the low-speed regime, whereas, with increasing speed in a tribometer setup, other parameters, such as surface adsorption kinetics due to continuous readsorption onto the surface after tribostress-induced desorption from the surface, start to play an important role in determining the efficacy of lubrication.^56,57^ Nevertheless, as shown in Figure 2, the overall Ψ values of PLL(20)-*g*-PEG(5) and PLL(20)-*g*-dex(10) were approximately comparable in this study. It is therefore reasonable to assume that the “tightly bound” water molecules contribute more to aqueous lubrication than the “loosely bound” molecules, and thus, the presence of “tightly bound” water molecules for the PEG chains only is likely to be responsible for the difference in the lubricating capabilities of the two copolymers.

The lower hydration capabilities of PLL-*g*-dex copolymers might also explain the need for both higher surface densities in the grafted hydrophilic chains and higher degree of overlap between them to achieve comparable antifouling capabilities with PLL-*g*-PEG copolymers, as shown in a previous study comparing the resistance to nonspecific protein adsorption of PLL-*g*-dex and PLL-*g*-PEG copolymers.^20^

## Conclusions

In this study, we have quantitatively characterized the hydration capabilities of surface-bound PLL-*g*-PEG and PLL-*g*-dex copolymers with varying structural features, including the molecular weight of dextran chains and grafting density of PEG or dextran chains on the PLL backbone. The areal solvation, Ψ, and number of water molecules per hydrophilic group, *n*_H_2_O/HG_, associated within the polymer layer were determined by combining QCM-D and OWLS measurements. Particular attention was paid to compare PLL(20)-*g*-PEG(5) and PLL(20)-*g*-dex(10) copolymers as they are most likely to generate comparable film thicknesses due to a comparable fully extended chain length. Both copolymers showed a highly stretched, “brush-like” conformation, as shown by the *L*/2*R*_g_ ratios being 0.46 to 0.41 for PEG(5) and 0.38 to 0.33 for dex(10) chains, respectively, over the varied grafting ratio, *g*, in this study. The dry mass and surface density of surface-grafted PEG(5) and dex(10) chains showed a local maximum at *g* = 6.5 (dex(10)) or 6.6 (PEG(5)) as a function of *g*, which results from the opposing trends of increasing molecular weight and PEG/dex chain density on PLL backbone vs decreasing adsorption onto the surface with decreasing *g*. Nevertheless, the wet mass and areal solvation, Ψ, showed a continuously increasing trend with decreasing *g*, which implies a continuously increasing hydrated PEG or dex film thickness with decreasing *g*. This was attributed to the increasing upward stretching of PEG/dex chains to relieve the increasing *local* steric repulsion between neighboring PEG/dex chains along a PLL backbone with decreasing *g*. Overall, the variation of Ψ with varying *g* was clearly higher for PLL(20)-*g*-PEG(5) than PLL(20)-*g*-dex(10) copolymers, and it reflects the stiffer characteristics of dex chains. Both copolymers showed a higher number of water molecules per hydrophilic group, i.e., *n*_H_2_O/HG_, compared to previous studies conducted on PEG or dextran chains in bulk solution, due to the higher density of monomer units on a surface and the dynamic nature of QCM-D in detecting surrounding water molecules. Nevertheless, *n*_H_2_O/HG_ was clearly higher for PLL(20)-*g*-PEG(5) copolymers than for PLL(20)-*g*-dex(10) copolymers, which is attributed to the presence of “structurally bound” or “tightly bound” water molecules, 2–3 per EG unit, present only for PEG chains. Thus, the slightly, yet clearly and consistently superior lubricating capabilities of PLL-*g*-PEG compared to PLL-*g*-dex copolymers with comparable structural features in previous studies can be best associated with the presence of such “tightly bound” water molecules exclusively for PEG chains rather than the total amount of water molecules within the polymer brushes detected by the combined approach of QCM-D/OWLS in this study.

## Supporting Information Available

The plots of dry mass, surface density of lysine (*n*_lys_), surface density of dex(5) chains (*n*_dex5_), wet mass, and areal solvation for PLL(20)-*g*-dex(5) and PLL(20)-*g*-dex(20); estimated distance between C atoms carrying an R group along a polypeptide; the measured average distance between PEG(5) or dex(10) chains on the surface; the experimentally determined average distance between PEG(5) or dex(10) chains on the surface, *L*, and the distance between PEG(5) or dex(10) chains on a PLL backbone in the fully stretched configuration, *L*′; the magnified plots of Figure 4 for *L*/2*R*_g_ for PEG(5) and dex(10) (PDF)
