# Supplementary material for: Quantitative Comparison of the Hydration Capacity of Surface-Bound Dextran and Polyethylene Glycol
Source: Langmuir. 2024 Jun 26;40(27):14130–40. doi: 10.1021/acs.langmuir.4c01582 (PMC11238585; doi:10.1021/acs.langmuir.4c01582)
Supplement: Supplementary file 1 — la4c01582_si_001.pdf [file la4c01582_si_001.pdf]

SUPPORTING INFORMATION

Quantitative comparison of the hydration capacity of surface-bound dextran and polyethylene glycol

Chiara Perrino,[a] *Seunghwan Lee*[a, b] and *Nicholas D. Spencer**[a]

[a] Laboratory for Surface Science and Technology, Department of Materials, Wolfgang-Pauli-Strasse 10, ETH Zurich, CH-8093 Zurich, Switzerland

[b]Institute of Functional Surfaces, School of Mechanical Engineering, University of Leeds, Leeds, UK, LS2 9JT

*correspondence: e-mail: nspencer@ ethz.ch

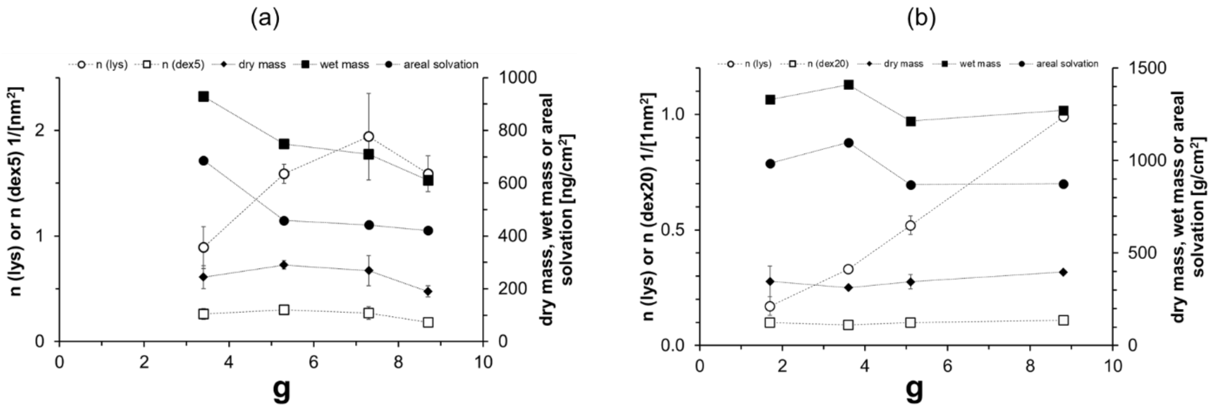

Figure S1. The plots of (a) dry mass, surface density of lysine (n_{lys}), surface density of dex(5) chains (n_{dex5}), wet mass, and areal solvation (ψ) for PLL(20)-*g*-dex(5) (b) dry mass, surface density of lysine (n_{lys}), surface density of dex(20) chains (n_{dex20}), wet mass, and areal solvation (ψ) for PLL(20)-*g*-dex(20).

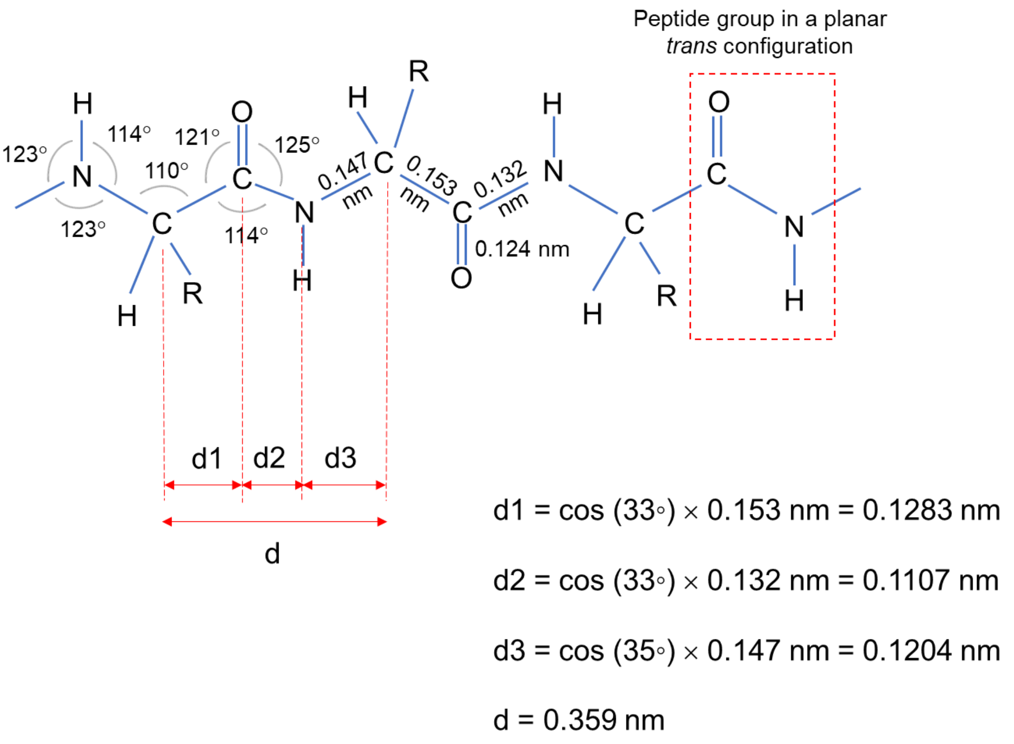

Figure S2. Estimated distance between C-atoms carrying R group along a polypeptide. The quantity of $d \times g$ can represent the distance between grafted PEG or dex chains along a PLL-*g*-PEG or PLL-*g*-dex copolymers. The figure and the detailed dimensions therein are adapted from “Three-Dimensional Structure of Proteins, N.V. Bhagavan, Chung-Eun Ha, in Essentials of Medical Biochemistry, 2011.

Table S1. The measured average distance between PEG(5) or dex(10) chains on surface (estimated from the model of 2-dimensional hexagonal arrangement[18]), *L*, and the distance between PEG(5) or dex(10) chains on a PLL backbone in the fully stretched configuration (shown in Figure S2), *L'*, and the ratio between them, *L*/*L'*.

	g	***L***	***L'***	***L/L'***
**PLL(20)-*g*-PEG(5)**	3.0	2.47	1.08	2.29
	4.4	2.40	1.58	1.52
	6.6	2.29	2.37	0.97
	11.2	2.61	4.02	0.65
**PLL(20)-*g*-dex(10)**	3.7	2.53	1.33	1.91
	4.8	2.69	1.72	1.56
	6.5	2.29	2.33	0.98
	8.6	2.61	3.09	0.84

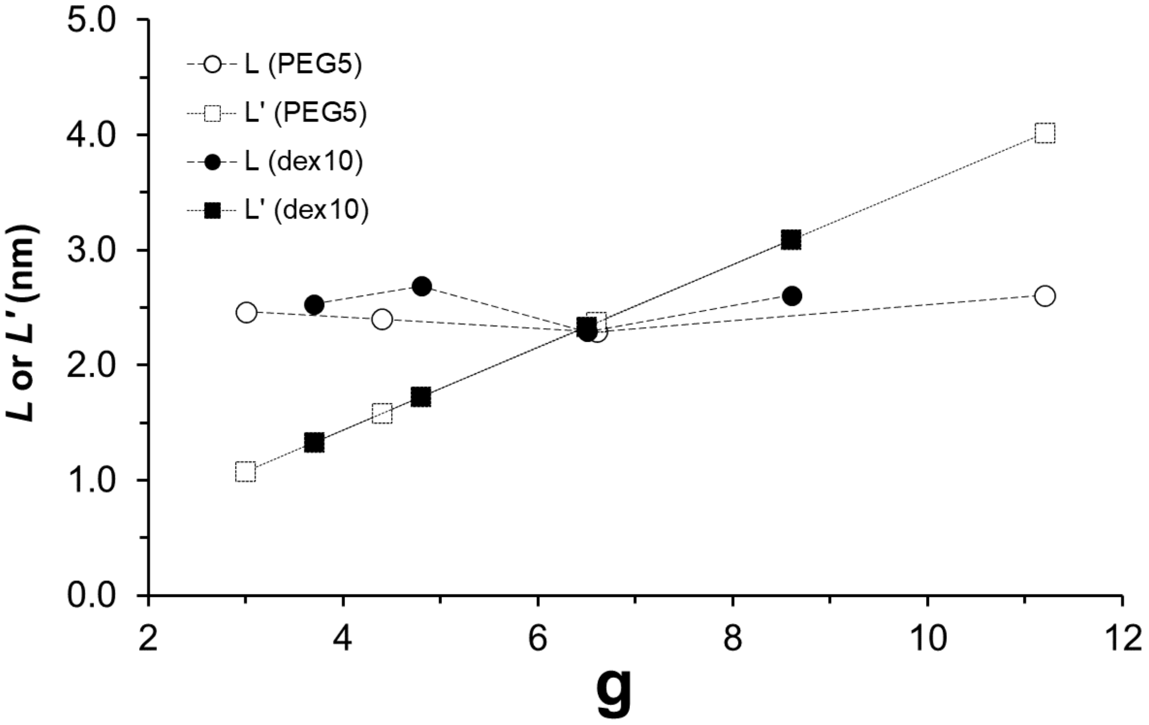

Figure S3. The plots of the experimentally determined average distance between PEG(5) or dex(10) chains on surface, *L*, and the distance between PEG(5) or dex(10) chains on a PLL backbone in the fully stretched configuration, *L'* (Table S1).

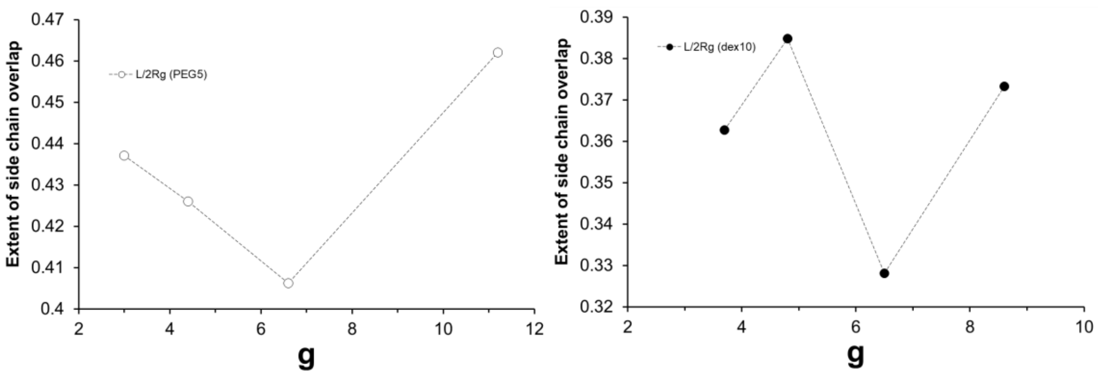

Figure S4. The magnified plots of Figure 4 for $L/2R_g$ (PEG5, left) and $L/2R_g$ (dex10, right).
